# Adiponectin‐expressing Treg cells in experimental thymic tumor model

**DOI:** 10.1111/1759-7714.14792

**Published:** 2023-01-05

**Authors:** Yuki Hanamatsu, Chiemi Saigo, Tamotsu Takeuchi

**Affiliations:** ^1^ Department of Pathology and Translational Study Gifu University School of Medicine Gifu Japan; ^2^ The United Graduate School of Drug Discovery and Medical Information Sciences Gifu University Gifu Japan; ^3^ Center for One Medicine Innovative Translational Research; COMIT Gifu University Gifu Japan

To the Editor,

The thymus plays a central role in the differentiation of T cell precursors to T regulatory cells (Tregs), which suppress autoimmune responses.^1^ Normal thymic architecture is critical to educate T lymphocytes, thus thymic tumors are often associated with various paraneoplastic autoimmune diseases, including myasthenia gravis, Hashimoto's thyroiditis, and/or even autoimmune diabetes.^2^

Nonobese diabetic (NOD) mice have been found to spontaneously develop autoimmune diabetes, and moreover they are prone to other autoimmune diseases.^3^ Curiously, NOD background is superior at generating Tregs in the thymus.^4^ Are these Tregs dysfunction in NOD related to autoimmune disease? Tregs from thymic tumor arising from NOD background mice may be useful to resolve the issue.

We previously reported a murine model of thymic tumor composed of multinodular thymic tumor with abundant lymphoid stroma in NOD strain background mice.^5^ We subsequently examined the phenotype of lymphocyte of this tumor model and found that it consisted partly of adiponectin expressing CD4^+^CD25^+^FOXP3^+^ Tregs (Figure 1).

**FIGURE 1:**
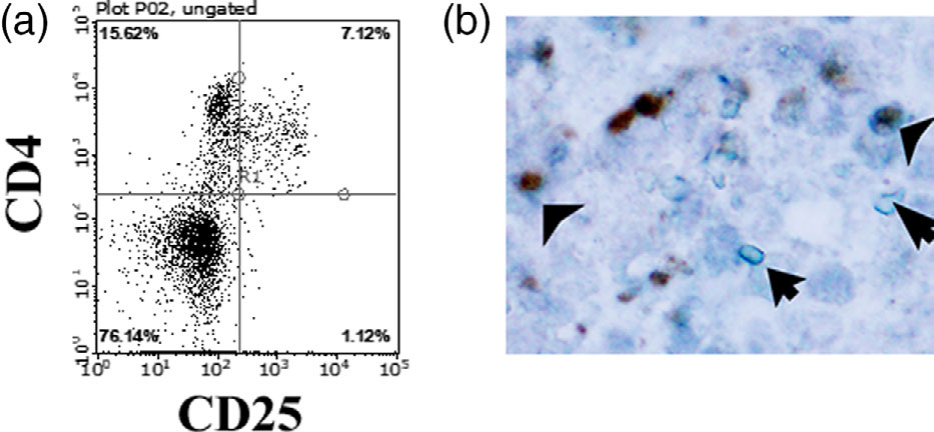
Representative immunostaining of lymphoid stroma. (a) Immunofluorescent cytostaining. Lymphoid cells partially exhibited staining on the cell surface with anti‐CD4 and CD25. Rat anti‐mouse CD4 (clone GK1.5) conjugated with phycoerythrin (PE) (catalog no. 1102040: Sony), while rabbit monoclonal antibody to mouse CD25 conjugated with FITC (catalog no. 50292‐M08H: Sino Biological Inc). In brief, cells were incubated with or without 1:100 diluted antibodies at 4°C for 1 h. After washing with phosphate‐buffered saline (PBS), the cells were analyzed with a Guava EasyCyte cell analyzer (Hayward). (b) Double immunohistochemical staining. Note the cytoplasmic adiponectin (blue staining) and nuclear FOXP3 immunoreactivity (brown staining) in murine model tissue sections. The arrow indicates the adiponectin positive but FOX3P^−^ lymphocyte, and the arrowhead indicates the adiponectin expressing FOXP3^+^ Tregs. For the detection of adiponectin and FOXP3, the tissues were formalin fixed and paraffin embedded, cut in section, incubated with anti‐adiponectin and FOXP3 antibodies, and stained using MACH 2 Double Satin 2 kit (Biocare Medical) as previously described.^6^ We used rabbit anti‐adiponectin antibody (cat. no. GTX107737, GeneTex Inc.) and mouse anti‐FOXP3 antibody (clone 3G3, Proteintech). Note that both antibodies are known to react with murine FOXP3.

Adiponectin is well characterized as an insulin‐sensitizing molecule. Recent advances based on a genetically engineered mouse model have unraveled that adiponectin‐expressing Tregs, which reside within the thymic nurse cell complexes, could play a role in obesity‐related metabolic diseases.^7^ In our three‐dimensional cell culture model, growth of adiponectin expressing Tregs was also dependent on direct contact with thymic epithelial cells, otherwise they declined to apoptosis.^5^ In other words, nursing of thymic epithelial cells is crucial to expand adiponectin expressing Tregs.

Our murine mode including three‐dimensional cell culture^5^ might be useful to understand the pathobiological property of adiponectin‐expressing Tregs in NOD background mice, which is a standard animal model for human type 1 diabetes.

## CONFLICT OF INTEREST

The authors declare no conflict of interest.
